# Repressive mutations restore function-loss caused by the disruption of trimerization in *Escherichia coli* multidrug transporter AcrB

**DOI:** 10.3389/fmicb.2015.00004

**Published:** 2015-01-22

**Authors:** Zhaoshuai Wang, Meng Zhong, Wei Lu, Qian Chai, Yinan Wei

**Affiliations:** Department of Chemistry, University of KentuckyLexington, KY, USA

**Keywords:** RND, oligomerization, membrane transporter, efflux pump, protein thermal stability

## Abstract

AcrAB-TolC and their homologs are major multidrug efflux systems in Gram-negative bacteria. The inner membrane component AcrB functions as a trimer. Replacement of Pro223 by Gly in AcrB decreases the trimer stability and drastically reduces the drug efflux activity. The goal of this study is to identify suppressor mutations that restore function to mutant AcrB_P223G_ and explore the mechanism of function recovery. Two methods were used to introduce random mutations into the plasmid of AcrB_P223G_. Mutants with elevated drug efflux activity were identified, purified, and characterized to examine their expression level, trimer stability, interaction with AcrA, and substrate binding. Nine single-site repressor mutations were identified, including T199M, D256N, A209V, G257V, M662I, Q737L, D788K, P800S, and E810K. Except for M662I, all other mutations located in the docking region of the periplasmic domain. While three mutations, T199M, A209V, and D256N, significantly increased the trimer stability, none of them restored the trimer affinity to the wild type level. M662, the only site of mutation that located in the porter domain, was involved in substrate binding. Our results suggest that the function loss resulted from compromised AcrB trimerization could be restored through various mechanisms involving the compensation of trimer stability and substrate binding.

## Introduction

*Escherichia coli* multidrug transporter AcrB and its homologues are the inner membrane component of the Resistance-Nodulation-Division (RND) family transporters in Gram-negative bacteria, which are major players in bacterial multidrug resistance (Blair and Piddock, 2009; Nikaido and Takatsuka, 2009; Nikaido and Pages, 2012; Zgurskaya and Nikaido, 2012). AcrB forms a tripartite pump system with membrane fusion protein (MFP) AcrA and outer membrane protein TolC. In the AcrAB-TolC complex, AcrB determines substrate specificity. The inward proton flow across the cytoplasmic membrane through a proton-relay pathway in the transmembrane domain of AcrB drives the active transport of substrates against their concentration gradient. In the process of substrate efflux, each AcrB monomer rotates through three conformations, access (or loose), binding (or tight), and extrusion (or open) (Murakami et al., 2006; Seeger et al., 2006, 2008; Vargiu and Nikaido, 2012).

**Figure 1:**
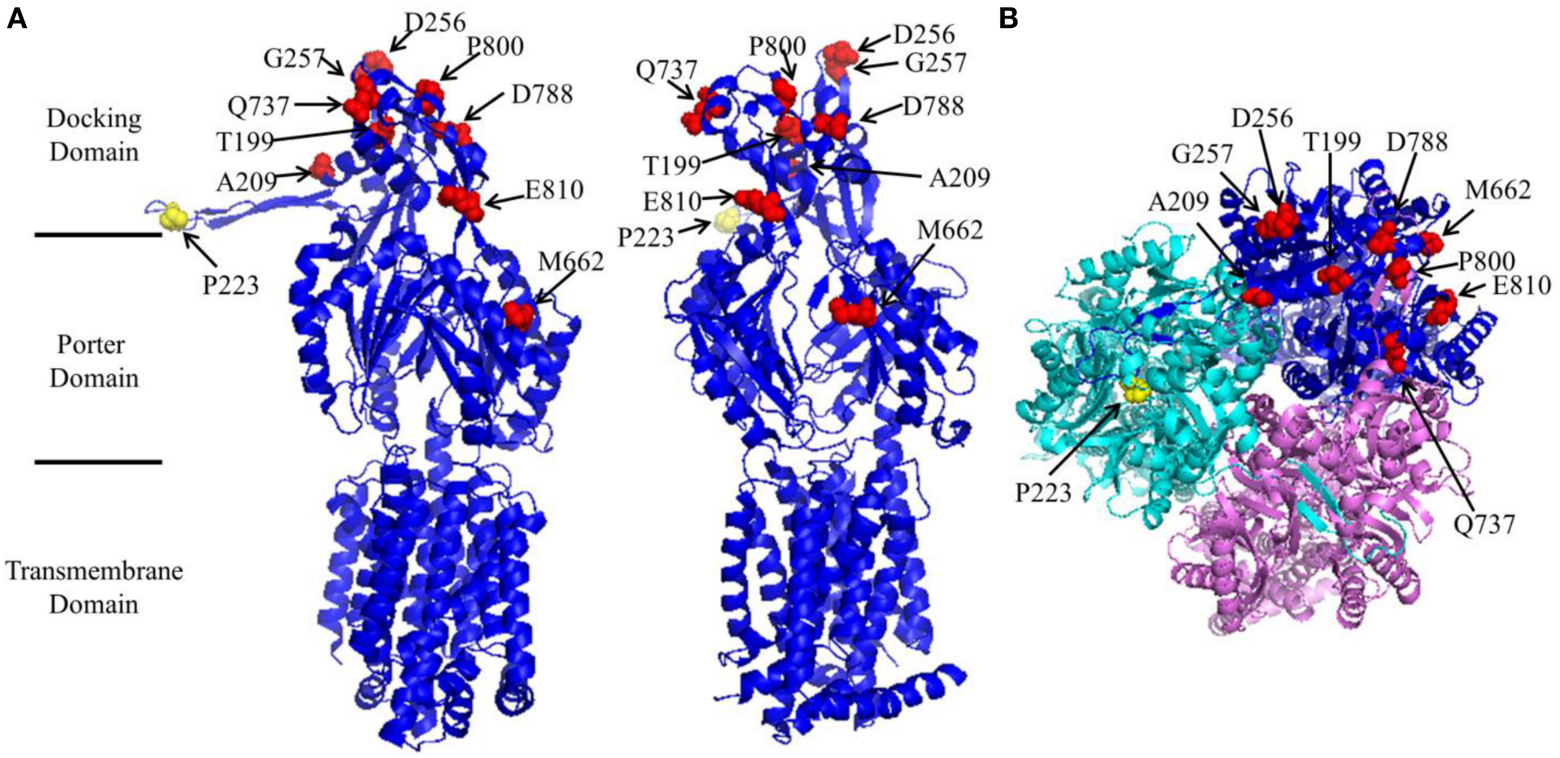
**Structure of AcrB (created using protein data bank file 1GIF using Pymol). (A)** Side view of a subunit from two different angles to reveal the location of Pro223 (yellow) and the suppressor mutations (red). Pro223 and suppressor mutation sites were labeled. The locations of different domains were marked. **(B)** Head view of AcrB trimer from the periplasmic side.

AcrB exists and functions as a homotrimer. Each subunit consists of 12 transmembrane helices (TMH), and two large periplasmic loops (LPL) which form a periplasmic domain. Mutations in the transmembrane domain, including D407A, D408A, K940A, and T978A, disrupt the proton relay network and disable the pump (Su et al., 2006; Eicher et al., 2009; Pos, 2009). The two LPLs exist in between TMH1 and TMH2 (LPL1) and TMH7 and TMH8 (LPL2). The periplasmic domain is further divided into a porter domain and a docking domain (Figure 1). Exchange of AcrB and AcrD periplasmic loops altered the substrate preference of the proteins, suggesting that residues dictating substrate specificity reside in the periplasmic domain (Elkins and Nikaido, 2002). A deep binding pocket was later defined through additional mutational and crystallographic studies (Yu et al., 2005; Sennhauser et al., 2006; Nakashima et al., 2011; Eicher et al., 2012). Depending on where they interact, substrates were divided into two groups: groove binder and cave binder (Bohnert et al., 2010; Takatsuka et al., 2010). Using Bodipy-FL-maleimide labeling, Nikaido and coworkers elucidated the entire substrate translocation pathway (Husain and Nikaido, 2010; Husain et al., 2011). More recently a switching loop was found to separate the binding location of different substrates into two sites, a proximal pocket and a distal pocket (Vargiu and Nikaido, 2012; Kobayashi et al., 2014). While large compounds bind to the proximal pocket before moving toward the exit, smaller substrates bind to the distal pocket.

Crystal structure of the entire AcrAB-TolC complex is not yet available. Two models of interaction have been proposed for AcrAB-TolC and similar tripartite transporters. A wrapping model was proposed first, in which the top part of the AcrB periplasmic domain interacts directly with the bottom of the periplasmic domain of TolC and AcrA wraps around the AcrB-TolC complex (Eswaran et al., 2004). Accordingly, the tip of the AcrB periplasmic domain is sometimes referred to as the TolC docking domain. More recently, a bridging model was proposed, in which the hexametric MFP forms a cylindrical bridge to connect the inner membrane protein and outer membrane protein (Xu et al., 2012; Du et al., 2014). In this bridging model, the outer membrane protein does not interact directly with the inner membrane protein.

The proper function of this delicate drug efflux machine requires the accurate assembly of the tripartite complex. While extensive studies have been done on the understanding of substrate binding, proton relay, and interaction between AcrB with AcrA/TolC, much less emphasis have been focused on the investigation of the trimerization of AcrB and how trimerization is correlated with other functional aspects such as interaction with AcrA/TolC and substrate binding (Lu et al., 2014). In each AcrB subunit, a long extended loop inserts into the neighboring subunit, which is important to the formation of AcrB trimer. We have previously created a well-folded monomeric AcrB mutant through truncating 17 residues from the loop (Lu et al., 2011). Later, we found that a single Pro223 to Gly mutation is sufficient to disrupt trimerization and lead to a drastic loss of activity (Yu et al., 2011). AcrB_P223G_ has low trimer affinity and its function can be partially restored through stabilizing the trimer via the introduction of an inter-subunit disulfide bond (Yu et al., 2011). To understand interactions that stabilize AcrB trimer, we conducted random mutagenesis experiments to identify suppressor mutations that restore functions to AcrB_P223G_. Nine single-site suppressor mutations were identified. These suppressor mutants were purified and characterized to investigate the potential mechanisms of function restoration.

## Materials and methods

### Random mutagenesis of AcrB and mutants screening

Random mutagenesis of AcrB was achieved by two methods, hydroxylamine hydrochloride treatment and error-prone PCR. Chemical mutagenesis by hydroxylamine hydrochloride was performed as described with slight modifications (Middlemiss and Poole, 2004). Plasmid pQE70-acrB_P223G_ was constructed in a previous study (Yu et al., 2011). pQE70-acrB_P223G_ was incubated in a potassium phosphate buffer (44 mM, pH 6.0) containing 5 mM EDTA and 0.46 M hydroxylamine hydrochloride at 70°C for 40 min. To quench the reaction, Tris-Cl (pH 8.0) and EDTA were added to the mixture to final concentrations of 90 and 9 mM, respectively. Treated plasmid was purified using a gel extraction kit (Qiagen, CA) and used for transformation.

Error prone PCR was carried out using GeneMorph II EZClone domain mutagenesis kit following the manufacturer's instruction (Agilent Technologies, Inc., Clara, CA). pQE70-AcrB_P223G_ was amplified using Mutazyme II DNA polymerase with two pairs of primers. Primers PP1 (5′-atgcctaatttctttatcgatcgc-3′) and PP2 (5′-AACGGCGTGGTG TCGTATGG-3′) were used to introduce mutations into the first large periplasmic loop of AcrB. Primers PP3 (5′-cgttgatcctgactccagctc-3′) and PP4 (5′- ATCGACCAGCTCTCGT ACAG-3′) were used to introduce mutations in the second large periplasmic loop. PCR products were separated and purified by gel electrophoresis. The purified PCR product was used as mega-primers and pQE70-AcrB_P223G_ was used as the template during the following EZClone reaction. DpnI was then added to digest the template plasmid.

Mutated plasmids generated through hydroxylamine treatment or error-prone PCR were used to transform *E. coli* BW25113*ΔacrB* through electroporation. The resultant cells were incubated with 1 mL LB broth with shaking at 37°C for 1 h before plated on LB agar plates containing 100 μg/mL ampicillin, 50 μg/mL kanamycin, and 16–32 μg/mL AcrB substrate erythromycin. Plasmids were extracted from colonies that were able to grow on the erythromycin plates and retransformed into BW25113*ΔacrB* strain. Drug susceptibility was tested. Plasmids that conferred decreased susceptibility were sequenced. Forty two suppressor clones were sequenced. Each mutations identified were further confirmed by introducing into P223G through site directed mutagenesis. Mutations yield false positive results were excluded. Nine unique single mutations that could restore P223G activity were identified. T199M were identified 14 times, D256N 6 times, M662I 2 times, D788K 2 times, and each of the other five mutations were only identified once. We have also observed the G223P back-to wild type mutation twice.

### Drug susceptibility assay of AcrB mutants

AcrB activities were examined using a drug susceptibility assay. The minimum inhibitory concentrations (MIC) of different strains were measured as described (Takatsuka and Nikaido, 2009). Plasmids encoding different AcrB constructs were used to transform BW25113*ΔacrB*. Freshly transformed cells were plated on LB-agarose plates containing 100 μg/mL ampicillin and 50 μg/mL kanamycin. The same ampicillin and kanamycin concentrations were used throughout the study. A single colony was used to inoculate LB media supplemented with ampicillin and kanamycin. The exponential-phase cultures of different strains were diluted to an OD_600nm_ unit of 0.1 using LB broth. 2 μL of this culture was spotted onto a series of LB-agar plate containing the indicated concentration of AcrB substrates. The agar plates were incubated at 37°C overnight and the lowest concentration of substrate that fully inhibited the bacteria growth was recorded as the MIC. BW25113*ΔacrB* strains transformed with plasmid-encoded wild type AcrB or AcrB_P223G_ were used as the positive and negative controls, respectively. Each experiment was repeated at least three times.

### Blue native page analysis of wild type and mutant AcrB

Protein purification was conducted as described (Lu et al., 2011). BN-PAGE analysis were performed as described (Wittig et al., 2006). A phosphate buffer (20 mM NaPi, 100 mM NaCl, 0.03% w/v DDM, pH 7.9) was used throughout the study unless otherwise noted. Purified protein samples were mixed with blue native loading buffer to reach a final concentration of 0.02 M 6-aminoocaproic acid, 1% dodecylmaltoside, 5% glycerol, 0.1% Coomassie brilliant blue G-250, pH 7.0. Protein samples were loaded to a 4–15% polyacrylamide gradient gel. Electrophoresis was performed using a running buffer (50 mM Tricine, 7.5 mM imidazole, 0.02% Coomassie brilliant blue G-250, pH 7.0) at 15 mA, in the 4°C refrigerator for 2 h. Protein bands were visualized after Coomassie blue stain. Band intensity was quantified using the software ImageJ.

### AcrA and AcrB interaction

pQE70-AcrB and its mutants were transformed into BW25113*ΔacrB* to study *in vivo* interaction between AcrB and AcrA. Dithiobis succinimidyl propionate (DSP) crosslinking and co-purification were performed as described with slight modifications (Zgurskaya and Nikaido, 2000). Cells were cultured overnight in 50 mL LB supplemented with ampicillin and kanamycin before harvested and washed with 5 mL phosphate buffer twice. The pellet was resuspended in 5 mL phosphate buffer containing 4 mM DSP, and incubated at 37°C for 30 min. Tris was added to a final concentration of 50 mM to quench the reaction. Proteins were purified using metal affinity chromatography as described (Yu et al., 2011). After elution, dithiothreitol (DTT) was added to the sample to a final concentration of 50 mM to reduce the disulfide bond in the DSP linker. Finally, the samples were subjected to SDS-PAGE and Western blot analyses using anti-AcrA antibody.

### Fluorescent labeling

BODIPY-FL-maleimide labeling was conducted following published method (Husain and Nikaido, 2010; Husain et al., 2011). BW25113Δ*acrB* cells harboring plasmids encoded AcrB mutants were cultured overnight at 37°C. Ten mL culture were harvested by centrifugation at 4000 × g for 5 min, washed twice using 10 ml of buffer A (50 mM potassium phosphate, 0.5 mM MgCl_2_, pH 7.0), and resuspended in 5 mL of the same buffer containing 0.4% glucose and 6 μM BODIPY-FL-maleimide. The mixture was shaken at room temperature for 1 h. Next, Cells were harvested by centrifugation, washed with 5 mL buffer A supplemented with 0.4% glucose, and then washed again with 5 mL buffer A. AcrB was then purified normally and resolved using SDS-PAGE. Fluorescence images were collected using a Typhoon Phosphor Imager with the excitation wavelength of 488 nm. The gel was then stained with Coomassie blue stain and imaged under white light. Band intensities were quantified using ImageJ (Rasband, 1997-2012; Schneider et al., 2012).

### Structural characterization using circular dichroism (CD) and fluorescence spectroscopy

CD spectra of purified wild type AcrB and its mutants were collected as described (Lu et al., 2011). CPM was from Invitrogen (GRAND ISLAND, NY). The CPM reactivity experiment was conducted as described in literature with minor modifications (Alexandrov et al., 2008). Briefly, a DMSO solution of CPM was freshly made at a concentration of 4.0 mg/mL. It was diluted in phosphate buffer by 40-fold to make a work solution, which was kept on ice during the experiment. 30 μL of the CPM working solution was mixed with 570 μL phosphate buffer in which the final concentration of protein was 4 μM. The fluorescence signal excited at 387 nm and emitted at 463 nm was monitored upon heating. Fluorescence emission intensity was normalized against the maximum intensity. Each experiment was performed at least three times.

## Results

### Identification of single point suppressor mutations that restore activity to AcrB_P223G_

In a previous study we found that mutation of P223 into Gly reduced the activity of AcrB to a level close to that of the *acrB* knockout strain (Yu et al., 2011). P223 locates close to the tip of a long protruding loop critical for AcrB trimerization. The mutation had little effect on proteins structure except that purified AcrB_P223G_ migrated mainly as a monomer in BN-PAGE. In addition, the observed function loss could be partially restored by the introduction of a pair of strategically placed Cys residues and the formation of inter-subunit disulfide bond, indicating that the disruption of trimerization was a major cause of function loss in AcrB_P223G_ (Yu et al., 2011). In this study, random mutagenesis was conducted to identify suppressor mutations that restore function to AcrB_P223G_. Plasmid encoding AcrB_P223G_ was first subjected to random mutagenesis, and then transformed into BW25113Δ*acrB* and plated onto LB agar plate containing 16 or 32 μg/mL erythromycin. Erythromycin is a well-established AcrB substrate. Here we chose to use erythromycin mainly due to the large difference of MIC between BW25113Δ*acrB* containing WT AcrB (128 μg/mL) and AcrB_P223G_ (8 μg/mL).

Two methods were used to introduce random mutations into AcrB_P223G_, hydroxylamine hydrochloride treatment and error prone PCR. The hydroxylamine hydrochloride method is biased, as it only results in transition from C to T and G to A (Middlemiss and Poole, 2004). Two suppressor mutations, T199M and D256N, were recovered using this method. The error-prone PCR method generated a more uniform spectrum of mutations with equivalent mutation rates. Two pairs of primers, PP1/PP2 and PP3/PP4 were used to introduce random mutations into two periplasmic regions: between residues 30–332 and residues 494–889. We focused our search in the two long loops that form the periplasmic domain. With this method, we rediscovered T199M and D256N, and seven additional single point suppressor mutations including A209V, G257V, M662I, Q737L, D788K, P800S, and E810K.

To confirm that the decreased drug susceptibility was actually caused by the restoration of AcrB activity, we introduced these suppressor mutations through site directed mutagenesis into plasmid pQE70-AcrB_P223G_ and transformed the resultant plasmids into BW25113*ΔacrB* for activity assay. Drug susceptibilities were measured for four AcrB substrates, including erythromycin, novobiocin, tetracycline, and tetraphenylphosphonium. BW25113*ΔacrB* transformed with pQE70-AcrB, pQE70-AcrB_P223G_, or the empty plasmid pQE70 were used as controls. MICs of the mutants and controls were shown in Table 1. For all AcrB substrates tested, the additional suppressor mutation greatly enhanced the activity of AcrB_P223G_.

**Table 1:** **MIC (μg/mL) of BW25113*ΔacrB* harboring the indicated plasmids**.

**Plasmids**	**MIC (μg/mL)**			
	**Ery**	**Nov**	**Tet**	**TPP**
pQE70-AcrB	128	320	2.56	640
pQE70-AcrB_P223G_	8	40	0.64	20
pQE70-AcrB_P223G/T199M_	128	160	2.56	640
pQE70-AcrB_P223G/A209V_	64	80	1.28	40
pQE70-AcrB_P223G/D256N_	32	160	2.56	320
pQE70-AcrB_P223G/G257V_	64	160	2.56	160
pQE70-AcrB_P223G/M662I_	128	80	1.28	160
pQE70-AcrB_P223G/Q737L_	64	160	2.56	160
pQE70-AcrB_P223G/D788K_	128	160	2.56	320
pQE70-AcrB_P223G/P800S_	64	80	1.28	80
pQE70-AcrB_P223G/E810K_	128	160	2.56	320

*Drugs tested were Ery, erythromycin; Nov, novobiocin; TPP, tetraphenylphosphonium; and Tet, tetracycline*.

A closer inspection reveals that there were several different MIC patterns for the nine suppressor mutants: (a) T199M, G257V, Q737L, D788K, and E810K restored the MIC to all four tested antibiotics to comparable levels. (b) For A209V, M662I, and P800S, the MICs to erythromycin were almost fully recovered (8-folds), but only limited effects were observed (2-folds) for the other three antibiotics. (c) For D256N, the MIC to erythromycin was only partially recovered (4-folds), but for other three antibiotics the recovery effect was quite obvious. To understand the mechanism of function restoration, we mapped the suppressor mutations (red) as well as P223 (yellow) onto the crystal structure of AcrB (Figure 1). Eight out of the nine repressor mutations located in the top part (docking domain) of the periplasmic domain. The only exception is M662, which is in the middle of the periplasmic domain (porter domain). Thus, eight out of nine suppressor mutations (other than M662I) are located distant from the substrate translocation pathway, and thus are not likely to be directly involved in interaction with substrates. These mutations might have led to subtle changes of the substrate translocation pathway through certain allosteric effect and subsequently resulted in the observed difference.

### Sequence alignment of suppressor mutations

To probe the potential role of these suppressor mutations, we aligned the *E. coli* AcrB sequence with 13 homologues. Table 2 showed the residues occupying these locations in all sequences together with their consensus score (Waterhouse et al., 2009). Substitutions identified in the suppressor mutation were also included. Although overall the sequence of AcrB is highly conserved among Gram-negative bacteria, most suppressor mutation sites were poorly conserved as revealed by the low consensus scores. This is a reasonable observation since mutation of highly conserved sites by itself might cause function loss as they are likely to play important roles in structure and function.

**Table 2:** **Sequence alignment of AcrB with homologes at sites that restored AcrB_P223G_ activity**.

**Residue number**	**199**	**209**	**256**	**257**	**662**	**737**	**788**	**800**	**810**
AcrB*(E. coli)*	T	A	D	G	M	Q	D	P	E
Replaced by	M	V	N	V	I	L	K	S	K
AcrD (*E. coli*)	T	S	D	G	V	L	Y	S	G
AcrF (*E. coli*)	T	V	D	G	V	A	L	F	Y
MexB (*P. aeruginosa*)	T	A	D	G	M	A	W	F	Y
AcrB*(H. pylori)*	D	V	I	G	V	Q	E	R	P
AcrB(*Y. pestis*)	T	I	D	G	V	A	W	F	Y
AcrB(*V. cholera*)	V	S	G	D	Q	I	W	Q	E
AcrB(*K. pneumonia)*	T	A	D	G	V	A	W	F	Y
AdeB (*A. baumannii*)	L	R	T	N	P	D	G	Q	R
AmeB (*A. tumefaciens*)	T	Q	G	A	I	D	N	S	S
TtgB (*P. Putita*)	T	A	D	G	V	L	Y	S	G
CmeB (*C. coli*)	G	V	N	E	G	M	N	T	V
AmrB (*P. aeruginosa*)	A	H	G	G	N	M	E	K	I
MtrD (*N. meningitidis*)	A	N	S	N	V	G	V	T	T
Consensus score	3	2	3	4	4	3	2	2	0

*Replacement residues identified in suppressor mutants were also shown*.

### Effect of suppressor mutation on protein expression

To determine the effects of additional mutations on AcrB_P223G_, we first examined the expression level of these double mutants. Plasmid encoding each mutant was transformed into BW25113*ΔacrB* for protein expression under the basal condition. Membrane vesicles were then extracted from the same amount of cells and subjected to Western blot analysis using an anti-AcrB antibody. We have previous demonstrated that AcrB_P223G_ expressed at a similar level as the wild type AcrB (Yu et al., 2011). Here we found that all mutant except for AcrB_P223G/D256N_ expressed at levels similar to that of the wild type AcrB and AcrB_P223G_ (Figure S1). The expression level of AcrB_P223G/D256N_ was approximately three folds higher than AcrB_P223G_. The exact reason for the observed increase of expression level is not clear.

### Effect of suppressor mutation on trimer stability

**Figure 2:**
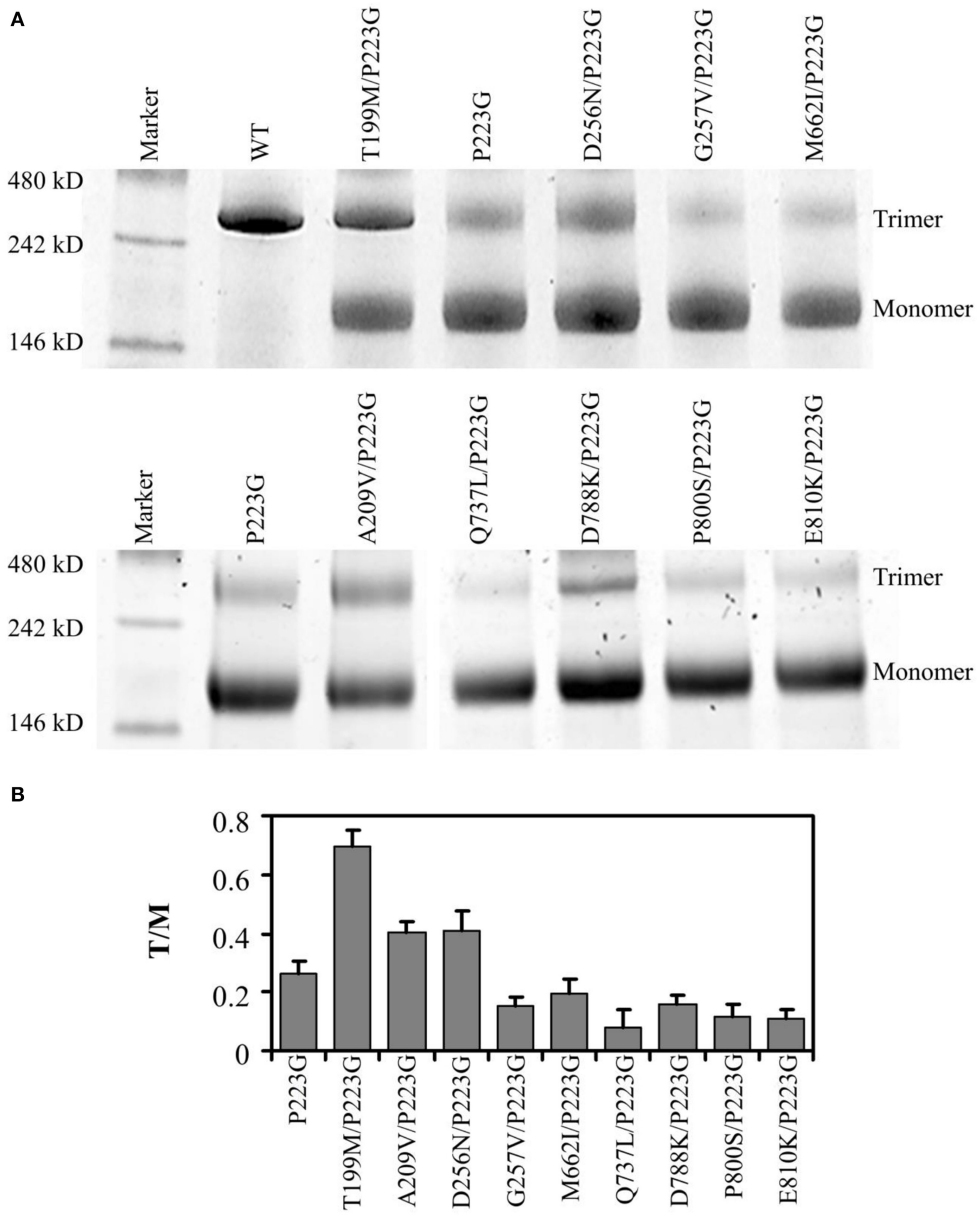
**Quarternary structure analysis. (A)** Representative images of BN-PAGE analyses of wild type (WT) and mutant AcrB. **(B)** Trimer to monomer ratio for each mutant was derived from the BN-PAGE analysis. Experiments were repeated three times. The average value and standard deviation were shown.

BN-PAGE was used to compare the trimer stability of each mutant. We have previously shown that while freshly purified wild type AcrB migrates predominantly as trimers, AcrB_P223G_ migrates predominantly as monomers (Yu et al., 2011). Figure 2A showed the representative gel images of the BN-PAGE results of the repressor mutants. AcrB_P223G_ and wild type AcrB were also shown as controls. Protein band intensities in the gel were quantified using ImageJ and trimer to monomer ratios were shown in Figure 2B. Two observations could be made: first, the trimer stability of all repressor mutants were much lower than that of the wild type AcrB; second, among the 9 suppressors, only three showed a significant increase of trimer stability, including AcrB_T199M/P223G_, AcrB_A209V/P223G_, and AcrB_D256N/P223G_. Other six mutations caused decreases in trimer stability as compared to AcrB_P223G_, especially in the case of AcrB_Q737L/P223G_. This result was a surprise since we expected the suppressor mutations to recover the MIC of AcrB_P223G_ through increasing the trimer stability.

### Interaction with AcrA

**Figure 3:**
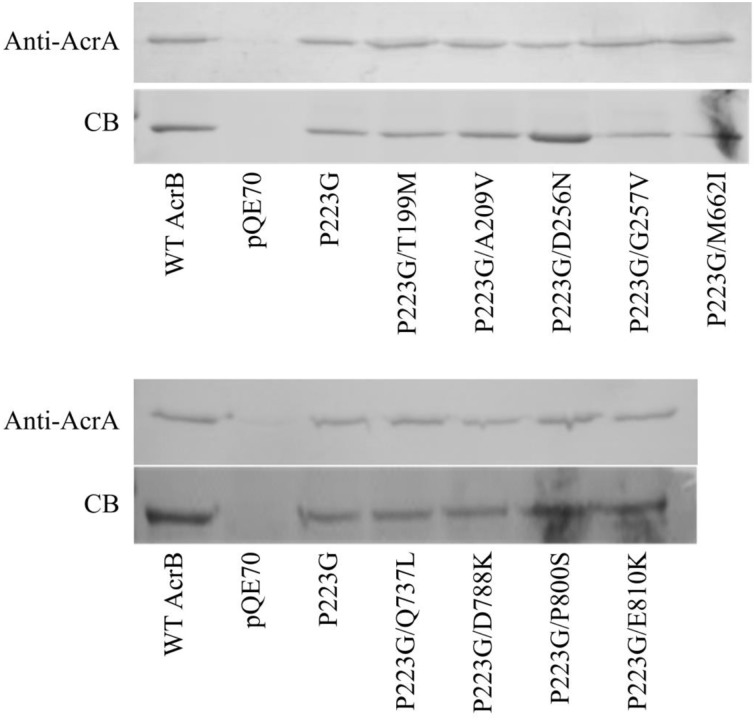
**Interaction with AcrA**. Anti-AcrA Western blot analysis (top panel) of co-purified AcrA from BW25113*ΔacrB* expressing wild type or mutant AcrB. The empty vector pQE70 was used as the negative control. The same gel was stained using Coomassie blue stain (bottom) to reveal that similar amount of AcrB was present in each sample.

The majority of mutations occurred at the top of the periplasmic domain in AcrB, the interaction site of AcrB with AcrA and/or TolC. A crosslinking protocol has been previously developed by Tikhonova et al. to demonstrate that AcrA and AcrB make direct contact (Zgurskaya and Nikaido, 2000; Tikhonova and Zgurskaya, 2004). To verify if these mutations have any impact on AcrA-AcrB interaction, we treated BW25113Δ*acrB* cells expressing different AcrB constructs with chemical crosslinker DSP, purified proteins using metal affinity chromatography, cleaved the disulfide bond in the crosslinker, and examined the quantity of AcrA co-purified with AcrB. The level of AcrA co-purified was revealed through anti-AcrA Western blot analysis, while the amount of purified AcrB was examined through staining a duplicate gel with Coomassie blue stain. Representative gel images were showed in Figure 3. BW25113*ΔacrB* transformed with pQE70 vector were used as a negative control to confirm that the signal was not from non-specific interaction of AcrA with the Ni-nitrilotriacetic acid (NTA) resin. Little signal could be detected for the negative control, suggesting AcrA did not retain on Ni-NTA resin without being cross-linked with AcrB.

The relative amounts of AcrA crosslinked to AcrB_P223G_ and wild type AcrB were similar, indicating that the P223G mutation, while decreased the trimer stability and compromised drug efflux activity, could still bind effectively to AcrA. The relative levels of AcrA cross-linked with all suppressor mutants tested were not significantly different from that of wild type AcrB or AcrB_P223G_. These results suggest that interaction with AcrA was not affected by the initial P223G mutation. Therefore, function restoration in the suppressor mutants was not likely through strengthening the interaction with AcrA.

### M662 is on the substrate translocation pathway

**Figure 4:**
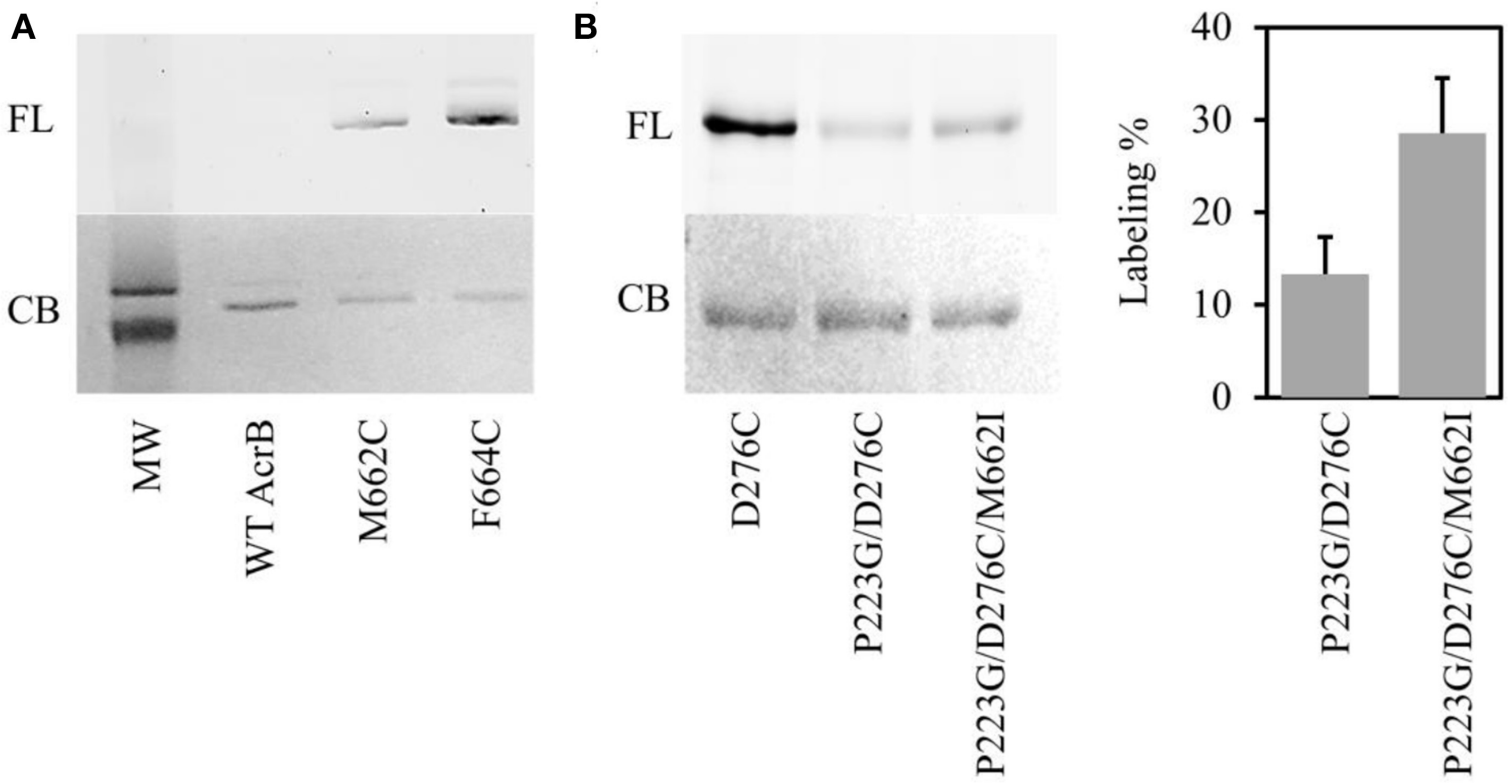
**Bodipy-FL-maleimide (FL) labeling of residues on the substrate translocation pathway. (A)** Labeling of WT AcrB, AcrB_M662C_, and AcrB_F664C_. The same gel was stained using Coomassie blue stain (CB). The two bands in the molecular weight marker are 120 kDa (top) and 85 kDa (bottom). **(B)** Labeling of AcrB_D276C_, AcrB_P223G/D276C_, and AcrB_P223G/D276C/M662I_. The same gel was stained using Coomassie blue stain (CB). The level of labeling in AcrB_P223G/D276C_ and AcrB_P223G/D276C/M662I_ was normalized to the level of labeling in AcrB_D276C_. The average value and standard deviation of three independent experiments were shown.

Of all suppressor mutants, AcrB_P223G/M662I_ is especially interesting. M662I is the only mutation that occurred in the AcrB porter domain. Nikaido and coworkers have developed a BODIPY-FL-maleimide labeling method to probe the substrate translocation pathway in AcrB periplasmic domain and proved that Phe664 and Phe666 were involved in substrate binding (Husain and Nikaido, 2010; Husain et al., 2011). In this method, a Cys was introduced at a target location. BODIPY-FL-maleimide selectively reacts with Cys introduced along the substrate translocation pathway. We speculate that Met662 was involved in substrate binding based on its location in the AcrB structure. To test the hypothesis, we conducted Bodipy-FL-maleimide labeling of a Cys introduced to replace M662 (Figure 4). Although there are two intrinsic cysteines in AcrB, both of them are distant from the translocation pathway. Thus, they were used as the negative control to confirm that BODIPY-FL-maleimide specifically label Cys at the substrate binding site. For positive control, we used AcrB_F664C_, a previously confirmed substrate-binding residue (Husain and Nikaido, 2010). As expected, no labeling was observed in wild type AcrB while AcrB_F664C_ was labeled well. AcrB_M662C_ could also be labeled by BODIPY-FL-maleimide, suggesting Met662 was indeed involved in substrate binding, most likely at the entrance of the drug translocation pathway. The relative level of labeling was weaker in AcrB_M662C_ as compared to AcrB_F664C_, suggesting that substrate interaction at this site was not as strong.

### M662I mutation improves substrate binding in the deep binding pocket of AcrB_P223G_

To investigate the potential effect of M662I mutation on substrate accessibility to the translocation pathway, we investigated the level of labeling of a previously verified site in the deep binding pocket, D276C (Husain and Nikaido, 2010; Lu et al., 2014). We performed BODIPY-FL-maleimide labeling in AcrB_D276C_, AcrB_P223G/D276C_ and AcrB_P223G/D276C/M662I_ (Figure 4B). The level of labeling was normalized to the labeling level in AcrB_D276C_. In AcrB_P223G/D276C_, the level of labeling was approximately 14 ± 4% of the level of labeling in AcrB_D276C_. The suppressor mutation, AcrB_P223G/D276C/M662I_, improved the labeling level to 28 ± 5%, approximately twice of the level in AcrB_P223G/D276C_. This result is consistent with the observed partial recovery of drug efflux activity in the repressor mutant.

### M662I mutation did not affect the overall structure nor improve the stability of AcrB_P223G_

**Figure 5:**
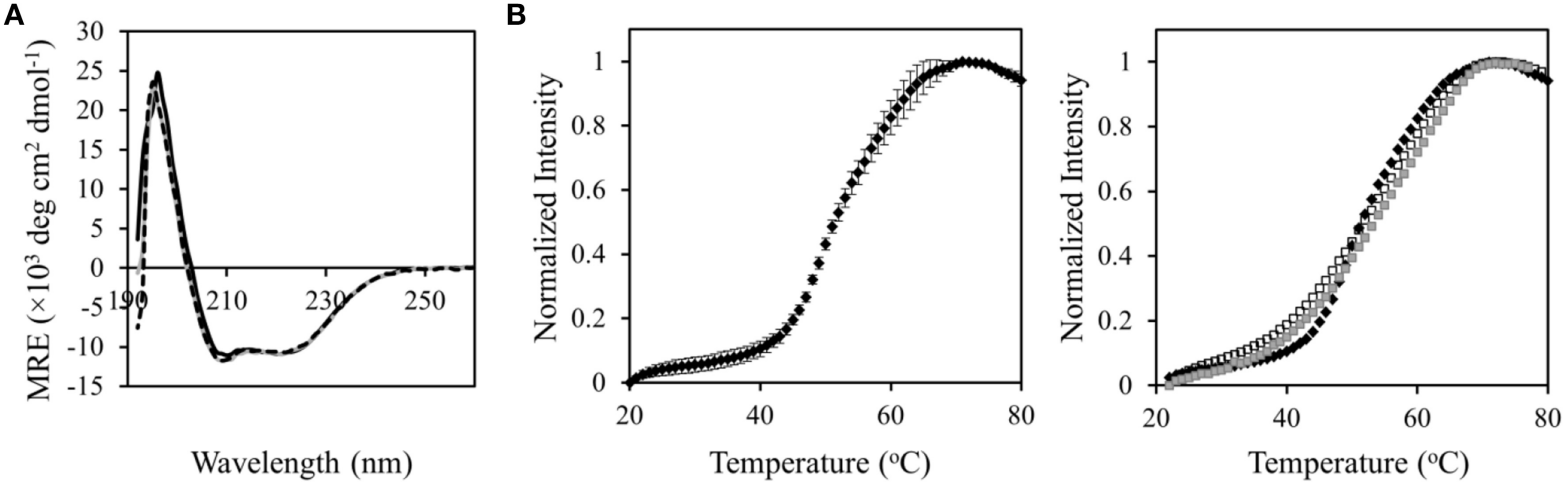
**Structural and stability characterization of AcrB_P223G_ and AcrB_P223G/M662I_. (A)** CD spectra of AcrB_P223G_ (black dotted) and AcrB_P223G/M662I_ (gray continuous) overlap with the spectrum of WT AcrB (black continuous). No observable change was induced by the mutations. **(B)** Thermal denaturation of WT (black diamonds), AcrB_P223G_ (open squares), and AcrB_P223G/M662I_ (gray squares) monitored using the reactivity with CPM (right panel). Fluorescence intensity was normalized to the maximum reading. Shown are the average values of three measurements. The average value and standard deviation for WT AcrB was shown as an example to reveal the reproducibility of the method (left panel).

To further elucidate the mechanism of function restoration, we compared the structure and stability of AcrB_P223G/M662I_ with those of AcrB_P223G_. CD spectra of both mutants overlap well on top of the spectrum of wild type AcrB, indicating that the overall conformation was not affected by P223G, nor the additional M662I mutations (Figure 5A). Next, we examined the stability of the proteins. In a previous study, we measured the thermal denaturation profiles of wild type AcrB and AcrB_P223G_ using a thermal denaturation method monitoring the CD signal at 222 nm (Yu et al., 2011). No significant difference was observed, indicating that either the two proteins have similar stability, or the technique is not sensitive to the intended measurement. Here we compared the stability of the proteins using a fluorescence labeling method (Alexandrov et al., 2008). Unfolding was monitored via the exposure of Cys residues upon heating and the subsequent reaction with N-[4-(7-diethylamino-4-methyl-3-coumarinyl) phenyl] maleimide (CPM). CPM is a thiol specific probe whose fluorescence emission intensity increased drastically upon reaction with a sulfhydryl group in a protein. The formation of a thioether and the concurrent attachment to the protein is revealed by a surge of fluorescence emission at 463 nm (excited at 387 nm). Since free Cys residues are usually found in the hydrophobic core of a folded protein structure, the unfolding of the protein exposes the Cys and greatly accelerates the thiol-specific reaction. The thermal denaturation of wild type AcrB revealed a fluorescence surge as approximately 52°C (Figure 5). The transition temperatures of the two mutants were similar as the transition temperature of the wild type protein, while the slopes were less steep, indicating a less cooperative unfolding behavior. This difference could be due to a decrease of trimer stability, as the thermal denaturation experiment monitors the global unfolding of the protein including trimer dissociation and protomer unfolding. The unfolding profiles of AcrB_P223G_ and AcrB_P223G/M662I_ were very similar, indicating that the restoration of function was not a result of improving structural stability.

## Discussion

Several factors are critical for the proper function for AcrB: drug binding, interaction with AcrA and TolC, proton relay through the transmembrane domain, and AcrB trimerization. In this study, we identified nine single suppressor mutations that could restore function to AcrB_P223G_. Trimer affinity of purified mutants was examined using BN-PAGE. Three mutants had higher trimer stability than AcrB_P223G_, while remained drastically lower than that of the wild type AcrB. For the rest of the mutant, trimer stability was not improved. They are likely to restore functions through alternative mechanisms. However, it is possible that *in vivo* in bacterial cells these mutations could enhance trimer stability as a secondary effect of other interactions, such as binding with AcrA/TolC or substrate.

The next factor we tested was the interaction with partner proteins. We chose to investigate the interaction with AcrA since AcrA and AcrB make direct contact according to both the wrapping model and bridging model, while TolC does not interact directly with AcrB in the bridging model. We found that AcrB_P223G_ and all suppressor mutants could be crosslinked with similar amount of AcrA as wild type AcrB, suggesting that the loss of function in AcrB_P223G_ was not likely an effect on disruption of AcrA interaction and the restoration of function was not an effect of improving interaction with AcrA.

Finally we focused on the study of one suppressor mutant, M662I, which is at the porter domain and close to the substrate translocation pathway. Using a fluorescent labeling experiment, we found that M662 was involved in substrate binding. Substrate binding in AcrB involves mainly hydrophobic interactions. Ile is slightly more hydrophobic than Met according to hydrophobicity scales generated by different studies (Rose et al., 1985; White and Wimley, 1999). It is interesting to notice that such a small change of hydrophobicity could significantly increase the MIC for all substrates tested. We further measured the level of labeling of residue in the deep binding pocket, D276C. We found that the M662I mutation doubled the relative level of labeling at this site on the P223G background. Finally, we confirmed that the additional M662I mutation did not induce an observable change on AcrB_P223G_ structure and stability.

While ligand-induced oligomerization has been observed in many receptor and signaling proteins, the ligand binding sites in these cases usually locate at the inter-subunit interface (Sigalov, 2010; Atanasova and Whitty, 2012). In AcrB, the substrate translocation pathway, including M662, is distant from the inter-subunit interface. Therefore, the potential effect on AcrB trimer stability, if any, has to be allosteric. Interaction between neighboring subunits in an AcrB trimer is clearly critical for function. During drug efflux each subunit adopts a different conformation and cycles through the three conformations in turn. Recently Pos and co-workers determined the structure of AcrB at high enough resolution to resolve unambiguously the side chains of critical residues involved in proton translocation in the transmembrane domain and rationalized how the cross-talk among protomers across the trimerization interface might lead to a more kinetically efficient efflux system (Eicher et al., 2014). Therefore, each subunit has to be able to “sense” the state of its neighbors and undergoes conformational change in a concerted manner. A key element differentiates the various conformational states is the mode of interaction with the substrate. Therefore, it is possible that improved interaction with substrate could enhance efflux activity either directly through more efficient uptake or indirectly via increasing trimer affinity. Interestingly, some ligands are found to enhance the transport of other ligands by AcrB, suggesting that ligand binding could potentially play a more active role in efflux (Kinana et al., 2013).

For the rest of the repressor mutant, it remains unclear how these mutations restored the function loss in AcrB_P223G_. Middlemiss and Poole conducted a random mutagenesis study to identify residues that are critical to the function of MexB, a close homolog of AcrB (Middlemiss and Poole, 2004). Most mutations that affected function were mapped to regions of MexB predicted to be involved in trimerization or interaction with MexA. In the same study, five suppressor mutations were identified to restore the activity of the MexB_G220S_, which was proposed to cause function loss via disruption of trimerization. Interestingly, similar as our observation in this study that all suppressor mutants identified, E796K, V203M/G581D, A737V, L738F, and D793N, locate in the docking area of the periplasmic domain. Protein-protein interaction during the assembly of the functional pump, including the intra-species interaction (trimerization of AcrB or MexB) and inter-species interaction with partner proteins (AcrA and/or TolC, MexA and/or OprM), seem to be correlated. The inter-species interaction may, to some degree, strengthen the intra-species interaction and compensate for function loss as a result of compromised trimerization. Finally, we have to acknowledge the limitation of the method we used to probe protein trimer stability using purified protein samples. It is possible that the suppressor mutations might have a more drastic effect on improving trimer stability in the cell membrane, and thus compensating for the loss of activity by directly improving the interaction between neighboring substrates in a trimer.

## Funding

We thank the National Science Foundation (MCB-1158036, Yinan Wei) and National Institute of Health (1R21AI103717, Yinan Wei) for supporting this work.

### Conflict of interest statement

The authors declare that the research was conducted in the absence of any commercial or financial relationships that could be construed as a potential conflict of interest.

## Abbreviations

R6G: Rhodamine 6G
PMSF: phenylmethylsulfonyl fluoride
WT: wild type
DDM: n-dodecylb-D-maltoside
NTA: nitrilotriacetic acid
RND: resistance-nodulation-cell-division
SDS-PAGE: sodium dodecyl sulfate polyacrylamide gel electrophoresis.
