# Supplementary material for: Repressive mutations restore function-loss caused by the disruption of trimerization in Escherichia coli multidrug transporter AcrB
Source: Front Microbiol. 2015 Jan 22;6:4. doi: 10.3389/fmicb.2015.00004 (PMC4303003; doi:10.3389/fmicb.2015.00004)
Supplement: Supplementary file 1 [file DataSheet1.DOCX]

Supplementary Material


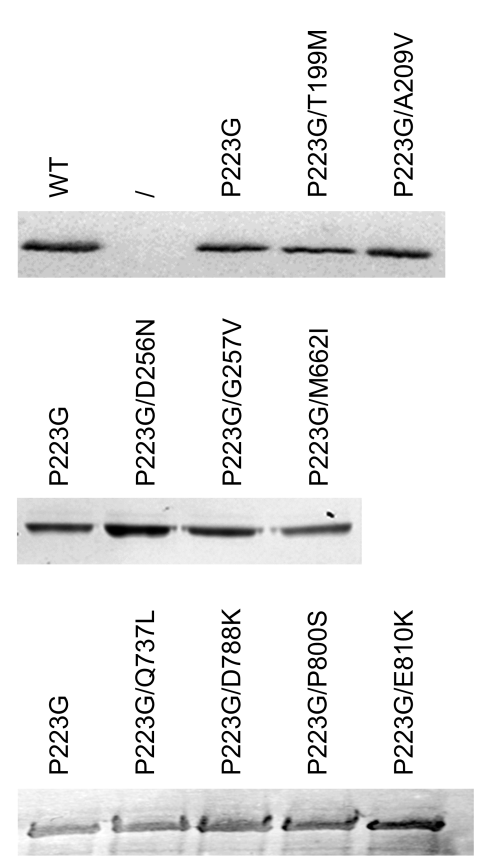


Figure S1. The representative image of anti-AcrB western blot showing the expression level of wild type AcrB, AcrB_P223G_, and nine repressor mutants. The negative control lane (/) was a sample prepared from BW25113*ΔacrB* containing the empty vector.
